# Predicting kidney failure from longitudinal kidney function trajectory: A comparison of models

**DOI:** 10.1371/journal.pone.0216559

**Published:** 2019-05-09

**Authors:** Jan A. J. G. van den Brand, Tjeerd M. H. Dijkstra, Jack Wetzels, Bénédicte Stengel, Marie Metzger, Peter J. Blankestijn, Hiddo J. Lambers Heerspink, Ron T. Gansevoort

**Affiliations:** 1 Department of nephrology, Radboud Institute for Health Sciences, Radboud university medical center, Nijmegen, The Netherlands; 2 Max Planck Institute for Developmental Biology, Tübingen, Germany, Center for Integrative Neuroscience, University Tübingen, Tuübingen, Germany; 3 CESP, Inserm, Univ Paris-Sud, UVSQ, Univ Paris-Saclay, Villejuif, France; 4 Department of Nephrology, University Medical Center Utrecht, Utrecht, The Netherlands; 5 Department of Clinical Pharmacy and Pharmacology, University Medical Center Groningen, Groningen, The Netherlands; 6 Department of Nephrology, University of Groningen, University Medical Center Groningen, Groningen, the Netherlands; University of Colorado Denver School of Medicine, UNITED STATES

## Abstract

**Rationale & objective:**

Early prediction of chronic kidney disease (CKD) progression to end-stage kidney disease (ESKD) currently use Cox models including baseline estimated glomerular filtration rate (eGFR) only. Alternative approaches include a Cox model that includes eGFR slope determined over a baseline period of time, a Cox model with time varying GFR, or a joint modeling approach. We studied if these more complex approaches may further improve ESKD prediction.

**Study design:**

Prospective cohort.

**Setting & participants:**

We re-used data from two CKD cohorts including patients with baseline eGFR >30ml/min per 1.73m^2^. MASTERPLAN (N = 505; 55 ESKD events) was used as development dataset, and NephroTest (N = 1385; 72 events) for validation.

**Predictors:**

All models included age, sex, eGFR, and albuminuria, known prognostic markers for ESKD.

**Analytical approach:**

We trained the models on the MASTERPLAN data and determined discrimination and calibration for each model at 2 years follow-up for a prediction horizon of 2 years in the NephroTest cohort. We benchmarked the predictive performance against the Kidney Failure Risk Equation (KFRE).

**Results:**

The C-statistics for the KFRE was 0.94 (95%CI 0.86 to 1.01). Performance was similar for the Cox model with time-varying eGFR (0.92 [0.84 to 0.97]), eGFR (0.95 [0.90 to 1.00]), and the joint model 0.91 [0.87 to 0.96]). The Cox model with eGFR slope showed the best calibration.

**Conclusion:**

In the present studies, where the outcome was rare and follow-up data was highly complete, the joint models did not offer improvement in predictive performance over more traditional approaches such as a survival model with time-varying eGFR, or a model with eGFR slope.

## Introduction

Chronic kidney disease (CKD) is a major public health issue, with an estimated worldwide prevalence of approximately 13%.[1] Persons suffering from CKD are at an increased risk of progression to end stage kidney disease (ESKD), hospitalization, and cardiovascular mortality.[2, 3] As kidney damage is often irreversible, treatment of CKD is aimed at preventing kidney function decline.[4] Prevention is likely to be most effective in patients with mild to moderate CKD who are at high risk of progression to ESKD.[5] To identify these patients accurate prediction of prognosis is needed. Most reported prediction models use survival regression on a snapshot of predictive factors taken at a single point in time.[6, 7] However, such models do not incorporate information about the evolution of important prognostic biomarkers, notably eGFR, and may thereby miss vital information about prognosis. Indeed, in clinical practice, physicians commonly use updated information from a series of consultations to make well informed therapeutic decisions.

Using longitudinal information to predict prognosis is intuitively appealing. Indeed, previous studies have combined a longitudinal linear model for kidney function decline with Cox regression.[8] In this work, the annual rate of kidney function decline assessed by the slope of estimated glomerular filtration rate (eGFR) was determined over a two-year training period using ordinary least squares linear regression. This eGFR slope was used as an additional prognostic factor in a Cox regression to predict progression to ESKD. However, others have shown that 10% to 40% of all CKD patients may show accelerated instead of linear eGFR decline.[9–11] Consequently, the impact of eGFR decline on prognosis may be underestimated. The use of eGFR as a time-varying covariate in a Cox regression may be of benefit in this case. This approach uses a step function to combine all the eGFR data of a patient to predict the probability of ESKD, and therefore no assumptions about the rate of eGFR decline are made. Both approaches require a training period to collect sufficient data for the model to be fitted. This may introduce immortal time bias. Patients with the most rapid progression may develop ESKD before the training period is over and are not included in the cohort used to develop the prognostic model. A solution to this problem may be through the use of joint models.[12] A joint model combines a longitudinal mixed model and survival model. Joint models do not have the limitations of the survival models described above, as they were initially developed to deal with informative drop-out in longitudinal mixed models. A common joint modeling approach in statistical literature is the shared parameter joint model.[13] and joint latent class model.

It is unclear which of the approaches described above results in the most accurate prediction of CKD progression to ESKD. Therefore, we reused data from two CKD cohorts, the MASTERPLAN and the NEPHROTEST studies, used as development and validation datasets, respectively, to compare the approaches using a pre-specified set of prognostic variables in patients with moderate CKD (eGFR > 30 mL/min per 1.73 m^2^).

## Methods

### Patient inclusion

The designs of the MASTERPLAN and NephroTest studies have been described previously.[14, 15] For the present study, we included 505 patients who participated in the MASTERPLAN study and had an eGFR >30 ml/min per 1.73m^2^ at baseline. We excluded patients who had received a kidney transplant prior to the study. The MASTERPLAN study was a randomized controlled trial evaluating intensified care with the aid of a nurse practitioner to standard care by a nephrologist (ISRCTN registry number 73187232).[14] Treatment was according to prevailing guidelines at the time and focused on blood pressure control, lipid reduction, reduction of proteinuria and promotion of a healthy lifestyle. We previously showed that the intervention group had lower blood pressure and less proteinuria during follow-up, ultimately resulting lower rates of renal progression.[16] In a subsequent causal analysis we showed that the effects of intensified blood pressure and anti-proteinuria treatment on renal outcome operated largely through eGFR.[17] Therefore, the prognostic impact of the treatment effect is captured by incorporating eGFR level and its decline into the prognostic prediction model.

For external validation, we used the NephroTest study, a prospective hospital-based cohort that enrolled 2084 adult patients with CKD at any stage, neither on dialysis nor living with a kidney transplant, who were referred by nephrologists to three physiology departments for extensive annual work-ups.[15] We excluded patients with CKD stage 4 or worse (eGFR <30 ml/min per 1.73m^2^, n = 635) and missing follow-up duration (n = 64) leaving 1385 patients for this analysis. Both studies were approved by an ethical committee (local IRB UMC Utrecht and CCTIRS MG/CP09.503 France) and all patients provided written informed consent.

### Variables and definitions

For each patient we recorded sex, age, race and diagnosis at the start of the respective studies. Diagnosis was coded as diabetic nephropathy, hypertension or vascular disease, glomerulonephritis, tubulo-interstitial nephritis, polycystic kidney disease, and other or unknown. Urine albumin to creatinine ratio (ACR) was also determined at baseline. Serum creatinine values were obtained at each visit, and standardized to a central laboratory in the MASTERPLAN study. The central laboratory used an isotope diluted mass spectrometry (IDMS) traceable enzymatic assay. In NephroTest plasma creatinine was measured using a modified kinetic Jaffe colorimetric method and a Konelab 20 analyzer (Thermo-Fisher Scientific) from the study start to 2008 and an enzymatic assay thereafter. Creatinine values obtained with the Jaffe assay were standardized to IDMS-traceable creatinine assay as previously described.[18] We calculated eGFR using the CKD-EPI equation for serum creatinine.[19]

### Outcome

**Fig 1:**
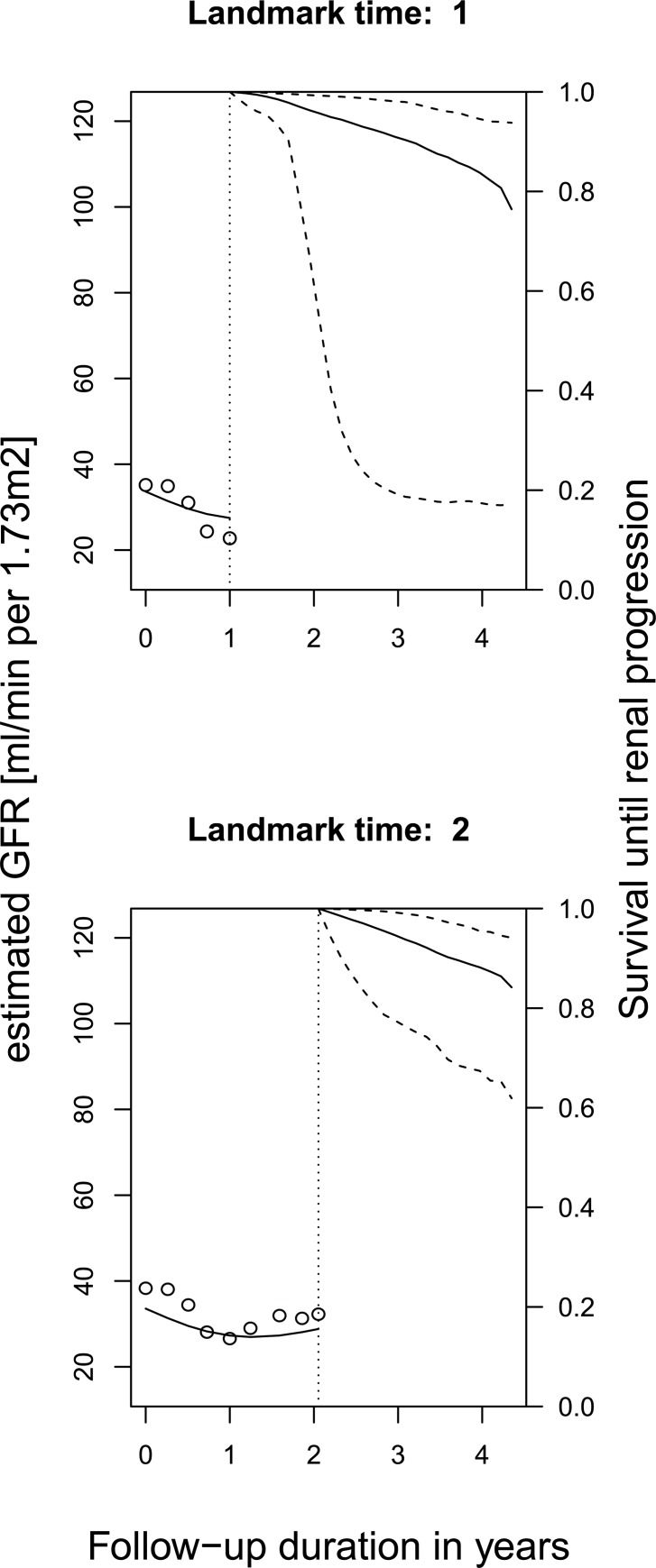
Dynamic prediction at various landmark times. Example of a dynamic prediction at landmark times 1, and 2 for time to end-stage kidney disease based on longitudinal trajectory of estimated glomerular filtration rate (kidney function) for patient in the MASTERPLAN cohort.

The outcome for the longitudinal model was eGFR slope over time until the landmark time. The outcome for the survival model was the occurrence of ESKD, defined as dialysis start, pre-emptive transplantation, death due to kidney failure, or an eGFR <15 ml/min per 1.73m^2^. For the all analyses, survival time was calculated from a landmark time of 2 years until a prediction horizon of 2 years (4 years total follow-up), as shown in an example for a single patient in Fig 1. The horizon indicates the maximum follow-up duration for which the models predict the risk of progression to ESKD. The landmark is the time since baseline during which a patient’s data is included in the prediction model. These values were chosen in for comparison to the well known Kidney Failure Risk Equation.[20]

Follow-up time was censored at ESKD, at the time of death for patients who died without reaching ESKD, or at four years (landmark + prediction horizon) if a patient did not suffer either event.

### Statistical methods

The following describes a brief overview of the modeling approach. For a more detailed description of the models and the R scripts that we used, we refer to the supplements.

We developed three different prognostic models the MASTERPLAN cohort and compared these to the Kidney Failure Risk Equation and each other in the NephroTest cohort: 1) a Cox regression that used baseline data and the current value of eGFR, 2) a Cox regression that in addition to the variables in the first model also included the eGFR slope determined from the first 2 years of follow-up in addition to baseline data, and 3) a joint model that used baseline data and eGFR data collected during the first 2 years of follow-up.

#### Model development

First, we specified a Cox regression model to predict onset of ESKD using the MASTERPLAN study. Covariates in this survival model were patient sex, age, baseline albuminuria, and the eGFR values at two years follow-up.

In the second approach, we included also the rate of eGFR decline determined over the first two years of follow-up in the model. The eGFR slope was estimated using separate ordinary least squares linear regressions for each individual patient using all eGFR measurement up to landmark time of two years.[8] Next, a Cox regression was fitted using covariate data, including sex, age, albuminuria, and current eGFR obtained at the landmark time with the addition of eGFR slope.

Third, we used a shared parameter joint model developed by Rizopoulos (R-package JM, version 1.4.5) to simultaneously estimate eGFR trajectory and survival until ESKD.[21] This approach assumes that eGFR trajectories within the CKD population form a heterogeneous mixture; *i*.*e*. the random effects joining the survival and mixed model process follow a Gaussian distribution. The mixed effects submodel included eGFR as the longitudinal outcome, and used follow-up time, sex, age, and baseline albuminuria as covariates. The survival submodel included sex, age, baseline albuminuria in addition to the current eGFR value and slope.

The joint model was also trained on the data available over the landmarking period between study start and 2 years follow-up and used to predict survival until ESKD two years later for a total follow-up of four years.

#### Model performance

We checked discriminative performance and calibration for the newly developed models and then compared them to the Kidney Failure Risk Equation. As the models were developed on the MASTERPLAN data, discriminative performance and calibration in that cohort are likely to be too optimistic compared to other settings. Therefore, we present only the performance in the NephroTest cohort. Discriminative power was evaluated using the area under the receiver-operating characteristic curve (ROC-AUC) (R-package timeROC, version 0.3).[22] In order to check calibration, we plotted the predicted probabilities for ESKD by observed ESKD status for each patient and fitted a calibration line using a LOWESS smoothed curve.

## Results

In total, 505 patients from the MASTERPLAN cohort were included at baseline. Patients had a median of 15 (IQR 11 to 22) serum creatinine measurements over the course of 4.76 years (SD ±1.1). Eleven percent (n = 55) of the patients reached ESKD during follow-up and 9% (n = 45) died. The NephroTest study cohort included 1385 participants who met the inclusion criteria, of whom 5% (n = 72) reached ESKD and 7% (n = 94) died. Creatinine measurements were less frequent in NephroTest patients as they were followed-up annually (median number of visits 2 (IQR 1 to 3)). Baseline characteristics were quite similar between the two studies, except the distribution of primary renal diagnoses which was slightly different (Table 1).

**Table 1:** Patient characteristics of the MASTERPLAN cohort and NephroTest cohort.

Characteristics	Baseline		Two year visit	
***MASTERPLAN* (n)**	**505**		**466**	
Males	69%		68%	
Age (years)	58.0	13.0	60.1	
Diagnosis				
Diabetic nephropathy	10%		10%	
Hypertensive or vascular nephropathy	27%		27%	
Glomerulonephritis	18%		18%	
Tubulo-interstitial nephritis	11%		11%	
Polycystic kidney disease	13%		12%	
Other or unknown	22%		23%	
eGFR-CKDEPI	50	18	48	20
UACR (mg/g)	68	(16–323)	55	(10–197)
***NephroTest* (n)**	**1385**		**394**	
Males	68%		67%	
Age (years)	58	15	58	15
Diagnosis				
Diabetic nephropathy	9%		9%	
Hypertensive or vascular nephropathy	23%		25%	
Glomerulonephritis	15%		19%	
Tubulo-interstitial nephritis	8%		12%	
Polycystic kidney disease	6%		7%	
Other or unknown	36%		29%	
eGFR-CKDEPI	51	18	48	19
UACR (mg/g)	50	(12–277)	68	(15–358)

UACR: urine albumin creatinine ratio, eGFR-CKDEPI: estimated glomerular filtration rate according to the CKD-EPI equation for serum creatinine,[19] Data are presented as proportions, mean and standard deviation, or median and 25^th^ and 75^th^ percentile.

In the Cox model that only used the current eGFR in addition to age, sex, and albuminuria, lower age and eGFR, and higher UACR, were strongly associated with progression to ESKD, but sex was not (Table A in S1 File). Similar associations were found in the Cox model that slope eGFR in addition to the current eGFR. Slope eGFR itself was associated with progression to ESKD, although the association was weaker compared to that between the current eGFR value and ESKD (Table B in S1 File). Male sex and baseline UACR were associated with eGFR during follow-up in the shared parameter joint model (Table C in S1 File). In the Cox submodel, current eGFR level, baseline UACR and the current slope of eGFR were associated with progression to ESKD, sex and age were not.

The area under the ROC curves (ROC-AUC) were largely similar for the Kidney Failure Risk Equation, Cox model with time-varying covariates, the Cox model with slope eGFR, and the joint model (Table 2).

**Table 2:** Discriminative performance in the NephroTest cohort.

Prognostic prediction model		ROC-AUC	95% confidence interval			P
Kidney Failure Risk Equation		0.94	0.86	-	1.01	ref
Cox model with time-varying eGFR		0.92	0.85	-	0.98	
Cox model with slope eGFR		0.95	0.90	-	1.00	
Shared parameter Joint Model		0.91	0.87	-	0.96	

ROC-AUC: Area under the receiver operating characteristic curve.

Discriminative performance according to the Kidney Failure Risk Equation, a Cox model with time-varying eGFR, aCox model with linear eGFR slope up the landmark time of 2 years, a shared parameter joint model, and a joint latent class model. Predictions were for progression to end-stage kidney disease within the next two years (*i*.*e*. horizon = 2) for a maximum of four years total follow-up.

**Fig 2:**
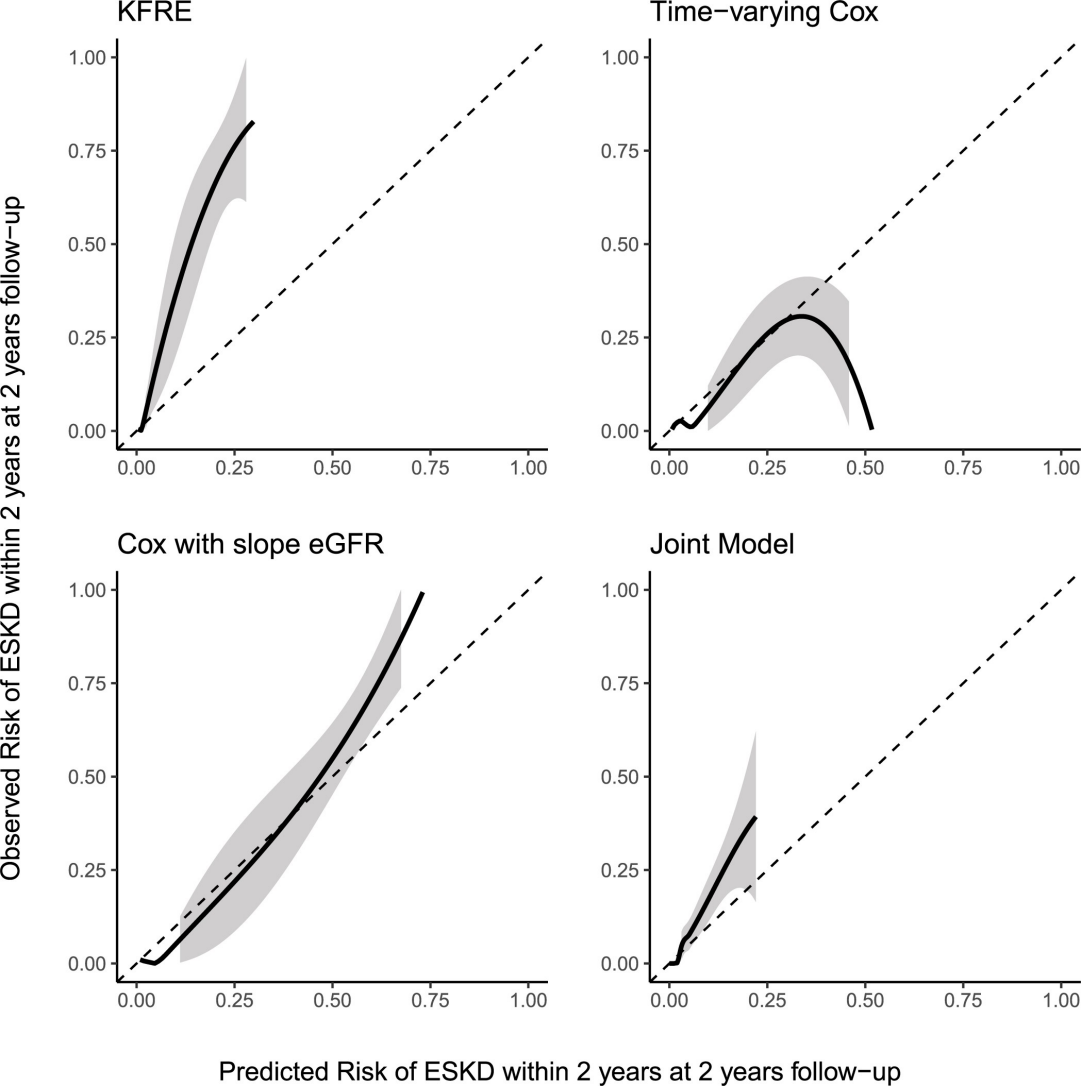
Calibration between predicted and observed risk of progression to end-stage kidney disease at five years follow-up in the NephroTest cohort. The black line indicates the average calibration line, the shaded area is the 95% confidence interval. The Kidney Failure Risk Equation (KFRE) was implemented without any re-estimation or recalibration. The other models were trained on the MASTERPLAN data and were used without re-estimation or recalibration to predict progression to end-stage kidney disease within the next 2 years (*i*.*e*. horizon h = 2) at 2 years of follow-up (*i*.*e*. landmark time t = 2).

Fig 2 shows the calibration between predicted and observed risk risk of progression to ESKD for the four models (including the KFRE) at a landmark of 2 year and a prediction horizon of 2 years (for al total follow-up of 4 years) in the NephroTest cohort. The Kidney Failure Risk Equation overestimed the risk of ESKD in this patient group. The Cox model that used current eGFR, and the joint model showed reasonable calibration in the low risk subjects, but tended to overestimate the risk in high risk subjects. The Cox model with slope eGFR showed the best calibration.

## Discussion

In the present study we have used three modeling approaches that incorporate follow-up values of eGFR to predict progression to ESKD in patients with CKD and a baseline eGFR >30 ml/min per 1.73m^2^ and compared these to the Kidney Failure Risk Equation, the best validated risk prediction model currently available. We used a Cox model that uses the most recent value of eGFR, a Cox model that included the linear eGFR slope in addition to the most recent eGFR,[8] and a shared parameter joint model that combines follow-up eGFR values and their slope.[21] We showed that discriminative performance was similar for the different approaches. The Cox model that included slope eGFR showed the best calibration with progression to ESKD at 2 years follow-up to a prediction horizon of 2 years.

To understand these results we need to consider the differences between the joint modeling approach and the ‘naive’ Cox models. The Cox models treat biomarker values measured over time as a step function. The hazard function varies as a step function accordingly. On the other hand, the joint model uses a mixed effects submodel to estimated the eGFR evolution as a continuous function over time. Likewise the hazard function follows a smooth function over time. We refer to the excellent work by Rizopoulos for a more detailed explanation.[23] As eGFR decreased linearly and gradually over time for most subjects in our study, and there are many follow-up measurements per subject, the step function used in the was a close approximation of the linear function. Therefore, the mixed effects submodel of the joint model offered only minor benefit is estimating eGFR evolution.

Furthermore, naive Cox approach assumed that all independent variables are exogenous. This means that the probability of observing the independent variables is not related to the outcome. If we were to consider a model for asthma attacks, we know that ambient air quality influences the risk of an asthma attack, but an asthma attack cannot influence ambient air quality. However, the fact that eGFR is available in our study, meant that a subject had not suffered an event prior to the time that the eGFR was determined. Hence, the exogeneity assumption was violated. Historically, the joint models were developed to deal with such informative drop-out in longitudinal data analysis, *i*.*e*. a violation of the exogeneity assumption. The exogeneity assumption may conceptually be compared missingness-completely-at-random (MCAR) in missing data analysis. The joint model does not assume exogeneity, and can be likened to a missing-at-random (MAR) assumption, meaning that missing values can be estimated from the observed data. From missing data analysis we know that if the number of missing values is small relative to the sample size, violation of the MCAR assumption will only have a minor impact on model performance. As the number of events was small in our study, violation of exogeneity assumption may have had only a minor impact on the predictive performance of a naive Cox models. Therefore, the more straightforward Cox model with slope eGFR showed the best performance.

### Relation to other studies

The present study was inspired by work of Li *et al*. and O’Hare *et al*.[10, 11] Patients in the MASTERPLAN and NephroTest studies had similar sex distribution, mean age, and mean baseline eGFR to the African American Study of Kidney Disease and Hypertension (AASK) cohort used by Li *et al*.[11] Both groups showed that eGFR decline does not necessarily follow a linear evolution over time. Thus using only the most recent GFR for prognostic prediction may not fully capture the true risk for an individual patient. Intuitively, physicians tend to look a eGFR evolution rather than only the most recent value. However, published prognostic prediction models tend to incorporate only cross-sectional data. We aimed to explore possibilities to incorporate longitudinal data, but were faced with the potential of non-linear kidney function decline. In order to determine eGFR trajectories, Li *et al*. developed a Bayesian smoothing technique that gives a probability distribution for the true underlying GFR slope. Theoretically, this approach is powerful, as it is robust to random variation due to measurement error and short term eGFR fluctuations. Unfortunately, incorporating the uncertainty from the Bayesian eGFR slope estimate into subsequent categorization of the eGFR trajectory is quite complex. Not including uncertainty surrounding the estimation of the eGFR trajectory may result in an underestimation of the standard error and too low p-values for the association between time-varying exposures and eGFR slope, when comparing periods of progression and non-progression within a single patient for example.[24] Furthermore, the approach by Li *et al*. may not fully account for the fact that patients with more rapid progression tend to have less eGFR data available, a phenomenon known as informative censoring, or missingness (not) at random.[21] Possibly the model formulated by Li *et al*. could be implemented in a joint model, combining the strengths of both approaches. This may be an interesting avenue for future research.

O’Hare *et al*. studied the eGFR trajectory two years prior to RRT initiation in a large cohort of veterans.[10] They used growth mixture model, a type of latent class analysis, to categorize patients based on their eGFR trajectory. In turn, they used these categories to predict mortality risk after initiation of renal replacement therapy. They identified four distinctive groups of patients based on eGFR trajectories. The first two groups had linear eGFR decline, although the first more gradual than the second. However, groups three (9% of the patients) and four (3%) had a non-linear eGFR trajectory, showing accelerating or even catastrophic decline. By only including patients who started renal replacement therapy, the authors avoided the problem of informative censoring. However, the results from the Veteran Affairs cohort may not apply to patients who do not reach renal replacement therapy.

### Strengths and limitations

Strong points of the present study include the use of a training cohort distilled from a clinical trial and validation of predictive performance in an external cohort. The prospective data collection according to the trial protocol ensured that our data was virtually complete in MASTERPLAN, only UACR was not determined at each follow-up visit. Likewise, the data collected in the NephroTest study was largely complete for all 4 variables. Moreover, serum creatinine values in both cohorts were standardized to isotope diluted mass spectrometry (IDMS). During the MASTERPLAN study, visits to the out-patient clinic were regular, every three months for most, and at least yearly for all. We pre-specified all prognostic factors to be included in the model, and used all models to predict at the same time point, and using the same data to exclude variation in data handling and to compare the model approaches *per se*.

Admittedly, our study does have limitations. First, the MASTERPLAN study was performed as a randomized controlled trial in a relatively young, predominantly Caucasian CKD population with a low eGFR for their age. This may mean that these were patients with more severe CKD than other populations. Moreover, the intervention has been shown to have an effect on blood pressure, urine albumin excretion and thereby on eGFR and the rate of renal progression during follow-up. Still the models performed well in the NephroTest cohort. Second, we aimed to predict outcome at a relatively early stage. In clinical practice prediction models should ideally be used timely, so that patients could be referred for ESKD care if needed. Therefore, we restricted the inclusion to patients with eGFR>30 ml/min per 1.73m^2^ at baseline. Consequently, we observed few events in both MASTERPLAN and NephroTestThe limited sample size may have impacted the predictive performance of the models, and thus the areas-under-the ROC curves are likely to be too optimistic. However, this holds for all the models and does not affect the comparison between modeling strategies. Arguably, the small sample size may have impacted model fitting. However, a rule of thumb of ~10 events per variable is commonly used for regression models like the Cox model that we fitted. We included 4 and 5 covariates in the respective Cox models for 50 events. Moreover, in a search of the literature we found simulations performed by Rizopoulos (Biometrics 2011) and Tsiatis and Davidian (Statistica Sinica 2004). They showed that the joint model performed well, even in small sample sizes. Third, we modeled estimated GFR and not measured GFR. Indeed, visual inspection of eGFR trajectories showed that many patients who reached ESKD had an unexpectedly upward sloping eGFR trajectory toward to end of their follow-up, possibly due to decreased muscle mass and therefore lower creatinine generation.[25] The inclusion of other markers for eGFR, such as cystatin C, may alleviate this issue. Fourth, the interpretation of regression parameters of the joint models is difficult. Even with the aid of plots and examples, it may be difficult to present the predictions and their possible consequences to a lay person. Fortunately, tools to communicate the results from dynamic predictions are available.[26] Finally, in the present study we used only a single longitudinal marker (eGFR) in our joint modeling approaches to inform the survival model. Incorporating other longitudinal markers such as UACR and blood pressure and allowing for the individual markers to be associated may further improve the performance of the joint modeling approaches.

### Meaning of the study and follow-up research

For the purpose of prediction progression to ESKD in patients with stage 3 CKD, a straightforward Cox model with a linear GFR slope seems appropriate. This was unexpected as patients with slower rates of progression are more likely to remain in the population at risk if drop-out prior to the landmark (the time at which the prediction is done) occurs. Such drop-out results in bias toward the null for any association between eGFR slope and outcome. Joint models have been originally developed to deal with informative drop-out in longitudinal data, and are therefore robust to such immortal time bias and missing data due to missed follow-up visits.[27] However, in our cohorts only few patients reached the endpoint, and consequently the proportion of “missing” eGFR slopes due to patients suffering the event prior to the landmark may be sufficiently small for the immortal time effect on the performance of the slope GFR model to be negligible. Moreover, we used data up to a landmark time of 2 years to predict the outcome within the next two years, comparable with the KFRE which we chose as a benchmark. The MASTERPLAN cohort and the NephroTest participants who had follow-up data available at two year had mostly complete eGFR data. This too allowed for the slope eGFR to be accurately estimated with OLS linear regression.

Therefore, we would recommend a straightforward survival model with GFR slope, estimated from a OLS regression model, when the outcome of interest is rare (*e*.*g*. <10%) and follow-up is highly complete. In these cases, bias due to informative drop-out is unlikely to have a substantial impact on predictive performance. In practice this means that a physician can simply use the most recent GFR value (or series of values when calculating a slope eGFR) to fill out a risk calculator. However, if the outcome is more frequent or more follow-up data is missing, a joint model may be a more valid alternative.

### Conclusions

In the present studies, where the outcome was rare and follow-up data was highly complete, the joint model did not offer improvement in predictive performance over more traditional approaches such as a survival model with time-varying eGFR, or a model with eGFR slope.

## Supporting information

S1 File Supporting methods and results. (PDF)
