# Supplementary material for: Predicting kidney failure from longitudinal kidney function trajectory: A comparison of models
Source: PLoS One. 2019 May 9;14(5):e0216559. doi: 10.1371/journal.pone.0216559 (PMC6508737; doi:10.1371/journal.pone.0216559)
Supplement: S1 File — (PDF) [file pone.0216559.s001.pdf]

# Supplements to: Predicting Renal Outcome in Chronic Kidney Disease from Longitudinal eGFR Trajectory: A Comparison of Model

## Contents


Statistical Methods ........................................................................................................................ 2

Baseline Cox model with time-varying eGFR ........................................................................ 2

Cox with slope eGFR ................................................................................................................ 2

Joint Model ................................................................................................................................ 3

Kidney Failure Risk Equation .................................................................................................. 4

Model performance .................................................................................................................. 4

ROC analysis ............................................................................................................................ 4

Calibration plots ........................................................................................................................ 4

Parameter estimates for the prognostic models ...................................................................... 6

# Statistical Methods

We developed three different prognostic models the MASTERPLAN cohort and compared these to the Kidney Failure Risk Equation and each other in the NephroTest cohort: 1) a Cox regression that used baseline data and the current value of eGFR as a time-varying covariate, 2) a Cox regression that in addition to the variables in the first model also included the eGFR slope determined from the first 2 years of follow-up in addition to baseline data, and 3) a joint model that used baseline data and eGFR data collected during the first 2 years of follow-up.

## Baseline Cox model with time-varying eGFR

First, we specified a Cox regression model to predict onset of ESKD using the MASTERPLAN study. Covariates in this survival model were patient sex, age, baseline albuminuria, and the eGFR values at two years follow-up. Estimated GFR was modeled as a time varying covariate. The Cox proportional hazards model with time varying covariates requires that a survival object with t_start, t_stop, and event is created. Clustering (*i.e.* multiple rows of observations per patient) can be accounted for within by using a robust sandwich estimator, see: [https://cran.r-project.org/web/packages/survival/vignettes/timedep.pdf](https://cran.r-project.org/web/packages/survival/vignettes/timedep.pdf). The R-code that was used to fit the model is shown below:

```
tvcox <- coxph(
  Surv(time.start, time.stop, event.tvcox) ~ male + I(egfr/5) +
  I(age/10) + log2(uacr+0.001) + cluster(pid),
  data = d, x= TRUE, na.action = "na.omit" )
```

## Cox with slope eGFR

In the second approach, we included also the rate of eGFR decline determined over the first two years of follow-up in the model. The eGFR slope was calculated using separate ordinary least squares linear regressions for each individual patient using all measurement up to landmark time of two years, as shown in the R code below:

First, we create a separate data.frame for each patient

```
list.patients <- split(d,d$pid)
```

We use a for loop to estimate a OLS regression slope for eGFR for each patient.

```
for (x in seq_along(list.patients)) {
```

We determine the exact landmark time point for each patient, as the data was not perfectly balanced.

```
  time.lm[[x]] <-
  list.patients[[x]]$time[abs(list.patients[[x]]$time - 2) ==
    min(abs(list.patients[[x]]$time-2))]
  list.patients[[x]]$time.lm <- time.lm[[x]]
```

Next, we only include follow-up until the landmark time, not any after.

```
   list.patients[[x]] <- list.patients[[x]][list.patients[[x]]$time
    <= list.patients[[x]]$time.lm,]
```

Then we obtain an OLS estimate for the eGFR slope using linear regression.

```
  lm.gfr <- lm(egfr ~ time, data = list.patients[[x]])
```

We remove the data prior to the landmark time,

```
  list.patients[[x]] <-
    list.patients[[x]][list.patients[[x]]$time==time.lm[[x]],]
```

and add the slope estimate to the data.frame for that patient,

```
  list.patients[[x]]$s.egfr <- lm.gfr$coefficients[2]
}
```

Finally, we join the data.frames of the individual patients to obtain a single data frame for analysis.

```
d.slope <- do.call(rbind, list.patients)
```

Next, a Cox model was fitted with the slope GFR as one of the predictors.

```
cox.slope <- coxph(Surv(time_event, event.cox) ~ male + I(egfr/5) +
I(age/10) + log2(uacr+0.001) + I(s.egfr/5), data = d.slope, x= TRUE)
```

## Joint Model

Third, we used a shared parameter joint model developed by Rizopoulos (R-package JM, version 1.4.5) to simultaneously estimate eGFR trajectory and survival until ESKD.[21] This approach assumes that eGFR trajectories within the CKD population form a heterogeneous mixture; *i.e.* the random effects joining the survival and mixed model process follow a Gaussian distribution. The mixed effects submodel included eGFR as the longitudinal outcome, and used follow-up time, sex, age, and baseline albuminuria as covariates. The survival submodel included sex, age, baseline albuminuria in addition to the current eGFR value and slope. The R-code for the model was as follows:

```
lme.jm <- lme(fixed = egfr ~ ns(time, df = 3) + male + I(age/10) +
  log2(uacr.bas+0.001), random = list(pid = pdDiag(form = ~
  ns(time,df = 3))), data = d.long, na.action = "na.omit")
```

For the joint model to work make sure that patients are included in both the longitudinal and survival dataset and the ordering must be the same as well.

```
surv.jm <- coxph(Surv(time_event, event.cox) ~ male + I(age/10) +
  log2(uacr.bas+0.001), data = d, x= TRUE)
```

Next, the first order derivatives are manually provided:

```
deriv.1 <- list(fixed = ~ 0 + dns(time, df = 3) ,
                random = ~ 0 + dns(time, df = 3),
                indFixed = 2:4,
                indRandom = 2:4)
```

Finally, the mixed and Cox model are joined using both time-updated eGFR value and eGFR slope:

```
jm <- jointModel(lme.jm, surv.jm,
```

```
                                            timeVar = "time",
                                            method = "spline-PH-aGH",
                                            parameterization = "both",
                                            derivForm = deriv.1)
```

The joint model was also trained on the data available over the landmarking period between study start and 2 years follow-up and used to predict survival until ESKD two years later for a total follow-up of four years.

## Kidney Failure Risk Equation

The Kidney Failure Risk Equation model was used as a comparator model. The calculation was taken from Tangri JAMA 2016 (Supplements) for a non-USA cohort:

```
pi.kfre <- -0.2201*( age/10-7.036) +
  0.2467*(male-0.5641) -  0.5567*(egfr/5-7.222) +
  0.4510*(log(uacr)-5.137)
S0t <- 0.9832
pred.kfre <- 1 - S0t^exp(pi.kfre)
```

## Model performance

We checked discriminative performance and calibration for the newly developed models and them compared to the Kidney Failure Risk Equation. As the models were developed on the MASTERPLAN data, discriminative performance and calibration in that cohort are likely to be too optimistic compared to other settings. Therefore, we present only the performance in the NephroTest cohort. Discriminative power was evaluated using the area under the receiver-operating characteristic curve (ROC-AUC) (R-package timeROC, version 0.3). In order to check calibration, we plotted the predicted probabilities for ESKD by observed ESKD status for each patient and fitted a calibration line using a LOWESS smoothed curve.

### ROC analysis

ROC analysis was performed using the predicted values from the respective model. Predictive values can be obtained using the predict functions for the individual models (available from the respective packages). For example:

```
pred <-predict(cox.slope, type = "expected", newdata = d.nt)
pred <- 1 - exp(-pred)
d.nt$pred <- pred

slope.roc<- timeROC(T=d.nt$time_event,
                    delta=d.nt$event.cox,
                    marker=d.nt$pred,
                    cause=1,
                    weighting="marginal",
                    times=4,
                    iid=TRUE
)
```

### Calibration plots

Calibration plots were obtained by plotting the predicted by the observed risk and fitting a loess smoother using the ggplot2() package.

```
p.slope <- ggplot(data = d.nt.cox, aes(x = 1-pred, y = event.cox)) +
  geom_smooth(color = "black") +
  geom_abline(slope = 1, intercept = 0,
    color = "black", linetype = 2) +
  theme_classic() +
  scale_x_continuous(limits = c(0,1)) +
  scale_y_continuous(limits = c(0,1)) +
  xlab("") +  ylab("") + ggtitle("Cox with slope eGFR")
ggsave("calibrate.slope.pdf", width = 5, height = 4)
```

## Parameter estimates for the prognostic models

Table A. Parameter estimates for the Cox model with time-varying eGFR in MASTERPLAN.

| Variable | ESKD HR | 95% CI |
|---|---|---|
| Male sex | 0.96 | 0.51 - 1.84 |
| eGFR (per 5 ml/min per 1.73m2) | 0.55 | 0.41 - 0.73 |
| Age (per 10yrs) | 0.92 | 0.75 - 1.14 |
| UACR at baseline per (per doubling) | 1.27 | 1.01 - 1.49 |

HR, hazard ratio. 95%CI, 95% confidence interval. eGFR, estimated glomerular filtration rate according to the CKD-EPIcr equation. UACR, urine albumin creatinine ratio in mg/g.

Table B. Parameter estimates for the Cox model with slope eGFR in MASTERPLAN.

| Variable | Landmark 1 year | |
|---|---|---|
| | HR | 95%CI |
| Male sex | 0.95 | 0.33 - 2.70 |
| eGFR (per 5 ml/min per 1.73m2) | 0.54 | 0.38 - 0.77 |
| Age (per 10yrs) | 0.86 | 0.63 - 1.17 |
| UACR (per doubling) | 1.14 | 0.95 - 1.37 |
| Slope eGFR (per 5 ml/min per year) | 0.74 | 0.55 - 1.00 |

HR, hazard ratio. 95%CI, 95% confidence interval. eGFR, estimated glomerular filtration rate according to the CKD-EPIcr equation. UACR, urine albumin creatinine ratio in mg/g. The Cox model was re-estimated at each landmark, as the slope eGFR may vary.

Table C. Parameter estimates for the shared parameter Joint Model in MASTERPLAN.

| **Variable** | | **95%CI** |
|---|---|---|
| *Longitudinal model* | **Beta** | |
| Intercept | 69.9 | 63.0 - 76.9 |
| Male sex | -14.2 | -17.2 - -11.2 |
| Age (per 10yrs) | -0.66 | -1.72 - 0.40 |
| UACR at baseline (per doubling) | -1.08 | -1.45 - -0.71 |
| Follow-up (years) | | |
| 0 to p33 | -0.82 | -2.43 - 0.79 |
| p33 to p67 | -3.80 | -5.68 - -1.91 |
| p67 to end | -1.97 | -3.07 - -0.87 |
| *Survival model* | **HR** | |
| Male sex | 1.11 | 0.54 - 2.28 |
| Age (per 10yrs) | 0.84 | 0.68 - 1.04 |
| UACR at baseline (per doubling) | 1.20 | 1.04 - 1.39 |
| eGFR current value (per 5 ml/min) | 0.93 | 0.90 - 0.96 |
| eGFR slope (per 5 ml/min per year) | 1.12 | 1.07 - 1.17 |

Beta, the parameter estimate for the longitudinal submodel reflects the difference compared to the population mean eGFR. HR, hazard ratio. 95%CI, 95% confidence interval. eGFR, estimated glomerular filtration rate according to the CKD-EPIcr equation. UACR, urine albumin creatinine ratio in mg/g. *Follow-up time was modeled using restricted cubic splines with knots placed a the 33rd and 67th percentile of follow-up duration.
